# ACTN1 supports tumor growth by inhibiting Hippo signaling in hepatocellular carcinoma

**DOI:** 10.1186/s13046-020-01821-6

**Published:** 2021-01-07

**Authors:** Qian Chen, Xiao-Wei Zhou, Ai-Jun Zhang, Kang He

**Affiliations:** 1 Reproductive Medical Center, Department of Obstetrics and Gynecology of Ruijin Hospital, School of Medicine, Shanghai Jiaotong University, 197 Ruijin 2nd Road, Shanghai, 200025 China; 2 Department of Liver Surgery, Renji Hospital, School of Medicine, Shanghai Jiaotong University, 160 Pujian Road, Shanghai, 200127 China

**Keywords:** ACTN1, Poor prognosis, Tumor growth, Hepatocellular carcinoma, Hippo signaling

## Abstract

**Background:**

Alpha actinins (ACTNs) are major cytoskeletal proteins and exhibit many non-muscle functions. Emerging evidence have uncovered the regulatory role of ACTNs in tumorigenesis, however, the expression pattern, biological functions, and underlying mechanism of ACTN1 in hepatocellular carcinoma (HCC) remain largely unexplored.

**Methods:**

Immunohistochemical analysis of a HCC tissue microarray (*n* = 157) was performed to determine the expression pattern and prognostic value of ACTN1 in HCC. In vitro loss-of-function study in HCC cells were carried out to investigate ACTN1 knockdown on cell proliferation. In vivo subcutaneous xenograft model and intrahepatic transplantation model were generated to decipher the contribution of ACTN1 in the tumor growth of HCC. Gene set enrichment analysis, quantitative real-time PCR, Co-immunoprecipitation, immunofluorescence and western blotting were performed to identify the underlying molecular mechanism.

**Results:**

It was found that ACTN1 was significantly upregulated in HCC tissues and closely related to llpha-fetoprotein level, tumor thrombus, tumor size, TNM stage and patient prognoses. Knockdown of ACTN1 suppressed in vitro cell proliferation and in vivo tumor growth of HCC cells. Mechanistically, knockdown of ACTN1 increased Hippo signaling pathway activity and decreased Rho GTPases activities. Mechanistically, ACTN1 could competitively interact with MOB1 and decrease the phosphorylation of LATS1 and YAP. The growth-promoting effect induced by ACTN1 was significantly abrogated by pharmacological inhibition of YAP with verteporfin or super-TDU.

**Conclusions:**

ACTN1 is highly expressed in HCC tissues and acts as a tumor promoter by suppressing Hippo signaling via physical interaction with MOB1. ACTN1 may serve as a potential prognostic marker and therapeutic target for HCC.

**Supplementary Information:**

The online version contains supplementary material available at 10.1186/s13046-020-01821-6.

## Background

Hepatocellular carcinoma (HCC) is a leading cause of cancer-related mortality worldwide [1]. HCC has a high mortality due to the high rate of postoperative recurrence and metastasis [2, 3]. Hepatitis B virus (HBV) and hepatitis C virus (HCV) infection have been well documented to be the main risk factors of HCC. Until now, hepatic resection and liver transplantation are the most curative therapeutic choices for HCC patients. However, the long-term survival rate remains poor because of the recurrence and metastasis after operation [4]. It was recognized that HCC development was affected by various intracellular signaling molecules and extracellular components [5, 6]. Therefore, it is of great importance to explore these molecular alterations and to identify therapeutic targets for this deadly disease.

Hippo signaling is a crucial regulator of organ development, tissue hemostasis and regeneration [7]. Dysregulation of the Hippo pathway has been characterzied in a variety of human cancers, including HCC [8–10]. Hippo signaling can be activated by many biological factors, such as: cell polarity or adhesion molecules, cellular metabolic status or other cellular signaling. Hippo signaling is initiated by a series of kinase cascades that lead to the regulation of two transcriptional coactivators, Yes-Associated Protein (YAP) and Tafazzin (TAZ) [11, 12]. Its core consists of the serine/threonine kinases mammalian sterile 20-like kinase 1 and 2 (MST1 and MST2) and large tumor suppressor 1 and 2 (LATS1 and LATS2). Firstly, MST1/2 and Salvador Family WW Domain Containing Protein 1 (SAV1) form a complex, and phosphorylate and activate LATS1/2. Then LATS1/2 phosphorylates and inactivates YAP/TAZ. YAP/TAZ loses its transcriptional activation of pro-proliferation and anti-apoptosis genes when it is restrained in the cytoplasm. By phosphorylation, YAP and TAZ are prevented from nuclear accumulation and interaction with transcription factors such as TEA transcriptional factor (TEAD)-1, TEAD2, TEAD3 and TEAD4 to prime transcriptional activity [13, 14].

Actinin is a kind of cytoskeletal molecules that belongs to the actin filament cross-linking proteins [15]. Actinins participate in cytokinesis by balancing the contraction of myosin II, then form a contractile ring with the latter [16]. In muscle cells, actinins are crucial in the linking of adjacent sarcomeres together through thin filaments, thus coordinate muscle contraction. Actinins also format and disassemble cell-matrix adhesion through the activation of phosphoinositide 3- kinase (PI3K), or build different cell to cell adhesions by working with integrins and intercellular adhesion molecules (ICAMs) [17, 18]. Recent reports indicated that there are four isoforms of actinin in mammals, named as ACTN1-ACTN4 respectively. ACTN1 is crucial for glioma cell motility, and also plays an important role in the progression of lung adenocarcinoma [19, 20]. ACTN2 and ACTN3 can interact with the parafibromin, which is involved in the hypermethylation and suppression of many oncogenes [21]. High expression of ACTN4 is associated with the poor prognosis in many types of cancers and contributes to diverse oncogenic activities [22–24]. However, the biological functions and underlying mechanism of actinin in HCC remain unclear until now.

In this study, we found that ACTN1 was significantly upregulated in HCC tissues and closely related to tumor size, TNM stage or patient prognoses. Genetic silencing of ACTN1 suppresses HCC cell proliferation and tumor growth. Furthermore, mechanism studies reveal that the effects of ACTN1 on the biological behaviors of HCC cells are dependent on the signaling of MOB1-LATS1-YAP axis.

## Materials and methods

### Cell culture and reagents

Liver cancer cell lines of human, including HepG2, Huh-7, LM3, MHCC-97H, MHCC-97 L, SK-Hep1, SMMC-7721, SNU-423 and SNU-475 were obtained from Cell Bank of the Chinese Academy of Sciences (Shanghai, China). Liver cancer cells were cultured in a specific culture medium according to ATCC instructions, supplemented with 10% (v/v) fetal bovine serum (FBS, Gibco, Grand Island, NY, USA) and 1% (v/v) penicillin (100 U/ml) and astreptomycin (0.1 mg/ml). All of above cells were incubated in a humidified incubator under 5% CO_2_ at 37 °C. Verteporfin (VP, S1786) and Super-TDU (S8554) were purchased from Selleck (Shanghai, China). Huh-7 cells were treated with 50 nM Verteporfin or 50 nM Super-TDU (1–31), respectively.

### Clinical HCC and non-cancerous liver tissues

Human HCC tissues and corresponding non-cancerous liver (NCL) tissues (*n* = 20) were all collected from Renji Hospital, Shanghai Jiaotong University, School of Medicine. The tissue microarray of human contained 157 HCC samples was generated at Renji Hospital, School of Medicine, Shanghai Jiaotong University. All samples were received from the patients who underwent surgical resection and signed informed consent before their operations. The clinicopathological parameters of the HCC patients from whom the samples were obtained. This research was approved by the Research Ethics Committee of the World Health Organization Collaborating Center for Research in Human Production (authorized by the Shanghai Municipal Government).

### Quantitative real-time PCR

Total RNA of HCC tissues or HCC cells was was isolated using TRIzol reagent (Invitrogen, Carlsbad, USA). Then 2 μg of RNA were reverse-transcribed into complementary DNA (cDNA) by using a PrimeScript™ 1st Strand cDNA Synthesis Kit (Takara, Japan) according to the manufacturer’s instructions. Resultant cDNA served as templates for PCR amplification by SYBR *Premix Ex Taq* (Takara) on a 7500 real-time PCR system (Applied Biosystems, USA). Primer sequences used in this study were shown as follows: ACTN1-F: TGAGGAGTGGTTGCTGAATGAG, ACTN1-R: AACTTCTCTGCCAGGTGGTCC; CTGF-F: TGGAGATTTTGGGAGTACGG, CTGF-R: CAGGCTAGAGAAGCAGAGCC; ANKRD1-F: GTGTAGCACCAGATCCATCG, ANKRD1-R: CGGTGAGACTGAACCGCTAT; CYR61-F: CCCGTTTTGGTAGATTCTGG, CYR61-R: GCTGGAATGCAACTTCGG. β-Actin was used as internal control for quantification. The data were analyzed by using the 2^−ΔΔCt^ approach.

### Immunohistochemical analysis

Paraffin-embedded sections were 5-μm thick sections, deparaffinized with xylene and ethanol, and then heated in 0.1 mol/L citrate buffer (pH 6.0) by microwaving for 15 min. After being cooled at room temperature, the sections were incubated in 3% hydrogen peroxide for 20 min to block endogenous peroxidase. Then, the sections were blocked with 10% (v/v) BSA (Sangon, Shanghai, China) to inhibit non-specific binding, followed by incubation with primary antibody for ACTN1 (Abcam, ab50599) at 4 °C overnight with a dilution at 1:200. After washing with PBS for three times, sections were incubated with secondary reagent. All the slides were labeled with DAB substrate liquid (Cell signaling Technology, USA) and counterstained by hematoxylin, and photographed with a microscope (Carl Zeiss, USA). Scoring was conducted according to the intensity of positive staining and the proportion of stained tumor cells (score 0: 0–5%, 1: 6–30%, 2: 31–70%, and 3: > 71%. Scoring was evaluated by two independent investigators who were blinded to the clinical information. Disagreements were resolved by consensus.

### Western blotting analysis

HCC tissues or cells were lysed in using RIPA Lysis Buffer (Beyotime Co., Jiangsu, China) containing the protease inhibitors following the manufacturer’s protocols. The protein concentration was determined with a BCA kit (Beyotime Co., Jiangsu, China). Lysates from cells were separated by sodium dodecyl sulfate (SDS)-polyacrylamide gel electrophoresis, transferred onto polyvinylidene fluoride (PVDF) membranes (PerkinElmer, Boston, MA, USA). After blocking in PBS/Tween-20 containing 5% (v/v) BSA, the membranes were blotted with indicated antibodies at 4 °C overnight. Then the membranes were then incubated by species-specific secondary antibodies (LI-COR) separately for 1 h at room temperature. The signals were detected by an Odyssey infrared imaging system (LI-COR, Lincoln, NE) and quantified by ImageJ software. The following antibodies were used in this study: ACTN1 (Abcam, ab50599, diluted at 1:1000), phospho-LATS1 (Cell Signaling Technology, #9157, diluted at 1:1000), total-LATS1 (Cell Signaling Technology, #3477, diluted at 1:1000), phospho-YAP (Cell Signaling Technology, #13008, diluted at 1:1000), total-YAP (Cell Signaling Technology, #14074, diluted at 1:1000), and β-actin (Abcam, ab8227, diluted at 1:1000).

### Lentivirus production

ACTN1 ORF of human was subcloned into the pEZlv105 vector (GeneCopoeia, USA) to generate pEZ-lv105-ACTN1 plasmid. Virus packaging was performed in 293 T cells after transfection of pEZ-lv105-ACTN1 by using Lipofectamine 3000 (Invitrogen). Viruses were harvested at 48 h and 72 h respectively after transfection, and virus titers were determined. Target cells including Huh-7 and LM3 cells were infected with 1× 10^6^ recombinant lentivirus-transducing units in the presence of 6 μg/ml polybrene (Sigma-Aldrich, Shanghai, China).

### Cell viability assay

To determine cell viability, 2 × 10^3^ MHCC-97H, SNU-423, Huh-7 or LM3 cells were seeded into a 96-well plate per well. A premixed solution of 10% (v/v) Cell Counting Kit-8 (CCK8, Dojindo, Japan) was added into each well and incubated in a humidified incubator under 5% CO_2_ at 37 °C for 2 h. After incubation, the plate was taken and the optical densities of 24, 48 and 72 h was measured respectively by using a microplate reader (Thermo Scientific) at a wavelength of 450 nm.

### Plate colony formation assay

MHCC-97H, SNU-423, Huh-7 or LM3 cells with indicated genetic manipulation were seeded into in 6-well tissue culture plates at a density of 500 cells per well. After 10–14 days, the colonies were fixed in ethanol, stained with 0.1% crystal violet, and counted by two individuals.

### Migration assay

Transwell chambers (Millipore, USA) were used to determine cell migration. Briefly, 5 × 10^4^ MHCC-97H or SNU-423 cells in 200 μl serum-free DMEM were seeded in the upper chamber. Then, 600 μl culture medium containing 10% FBS was added to the lower chamber. After 24 h, the migrated cells were fixed and stained with 0.1% (w/v) crystal violet. Three randomly selected fields were photographed and the numbers were counted.

### Subcutaneous and orthotopically intrahepatic transplanted model

For generation of subcutaneous transplanted model, 2 × 10^6^ sh-ACTN1 or sh-control MHCC-97H cells were detached and suspended in 100 μl serum-free DMEM. The cells were subcutaneously injected into mice (male, 6 weeks old, *n* = 5 per group). For generation of orthotopically intrahepatic transplanted model, 2 × 10^6^ sh-ACTN1 or sh-control MHCC-97H cells 40 μl serum-free DMEM/matrigel (1:1) for each BALB/c-nu/nu mouse. Through a 1 cm transverse incision in the upper abdomen under anesthesia, the cells were injected into the left hepatic lobe of each mouse (male, 6 weeks old, *n* = 5 per group). All mice were sacrificed after 4 weeks, and the tumors and livers were taken and fixed with phosphate-buffered neutral formalin and prepared for standard histological examination. Subcutaneous tumor and liver metastases were detected by hematoxylin and eosin (H&E) staining. All of mice were manipulated and housed according to protocols approved by the Shanghai Jiaotong University Animal Care Commission. All animals received humane care according to the criteria outlined in the “Guide for the Care and Use of Laboratory Animals” prepared by the National Academy of Sciences and published by the National Institutes of Health.

### GTPase activation assay

GTPase activation was performed according to standard procedures as described elsewhere [25]. In brief, Cells were harvested in magnesium-containing lysis buffer and the lysates were sonicated for 5 s and centrifuged for 30 min at 18,000×g and 4 °C the Rho/Rac/Cdc42 Assay Reagent Kit (Thermo Scientific, USA) according to the manufacturer’s instructions. The primary antibodies used were anti-RhoA (Cell Signaling Technology, #2117, diluted at 1:1000), and anti-Rac1 (Millipore, 05–389, clone 23A8, diluted at 1:200), anti-Cdc42 (Cell Signaling Technology, #2462, diluted at 1:1000).

### F-actin staining

Indicated HCC cells were seeded in 6-well IBIDI plates at a density of 1 × 10^5^ cells/well. The next day, cells were fixed with 4% ice-cold paraformaldehyde in PBS for 20 min at 4 °C and permeabilized with 0.5% Triton X-100 for 5 min, followed by staining with FITC conjugated phalloidin (2 μg/ml, Sigma) for 20 min. DNA was visualized using DAPI. Finally, Actin filaments were visualized using confocal microscopy (Leica Microsystems, Germany).

### Co-immunoprecipitation

For intracellular immunoprecipitation, Huh-7 cell lysates transfected with HA-tagged ACTN1 or vector control were subjected to immunoprecipitation with anti-HA monoclonal antibody (Millipore) or control IgG. Then the immunoblotting with anti-MOB1 antibody was performed.

### Immunofluorescence analysis

MHCC-97H cells were seeded at 12-well U-Chamber (Ibidi, Germany), fixed with 4% paraformaldehyde for 15 min, and permeabilized with 0.05% (v/v) Triton X-100 for 1 min at room temperature. Then the cells were incubated with primary antibodies against ACTN1 (Abcam, ab50599, diluted at 1:100) or MOB1 (Cell Signaling Technology, #13730, diluted at 1:100) for 90 min, followed by an Alexa Fluor 594-conjugated anti-rabbit antibody and Fluor 488 conjugated anti-mouse antibody (Jackson). The nucleus was stained with DAPI (Sigma-Aldrich, Shanghai, China) and the immunoflurescene signals were captured using confocal-scopy (Carl Zeiss, USA).

### Statistical analysis

Values are expressed as the mean ± standard error of the mean. Statistical analyses were performed using SPSS 16.0 for windows. Survival curves were plotted using the Kaplan–Meier method and analyzed with the log-rank test. The association between ACTN1 expression and the clinicopathological parameters of HCC patients was evaluated using Pearson’s Chi-square test. One-way analysis of variance was used for comparison between groups. *P* < 0.05 was considered to indicate a statistically significant difference.

## Results

### Highly expressed ACTN1 predicts a poor clinical outcome in HCC patients

**Fig. 1:**
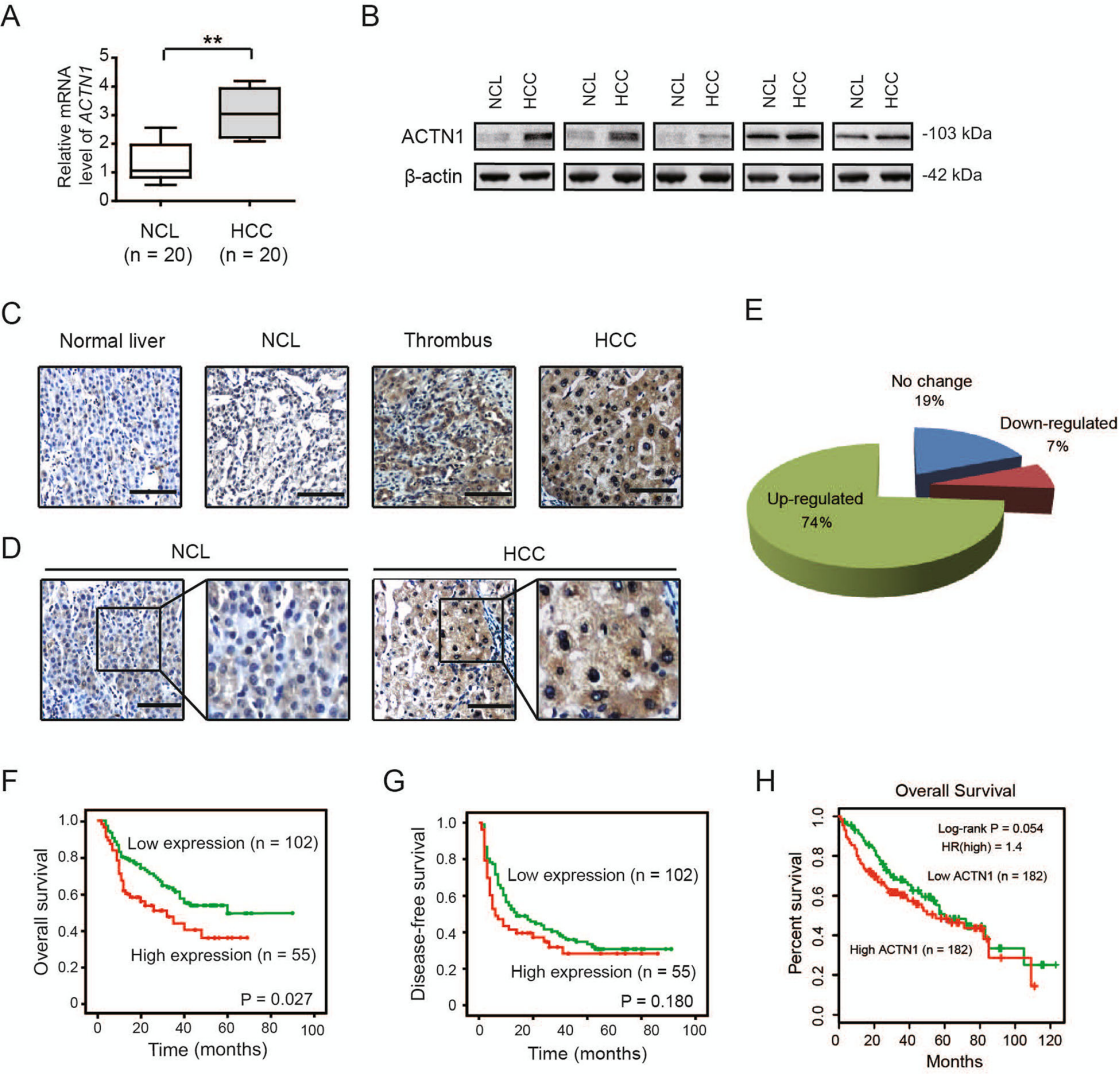
ACTN1 is highly expressed in HCC tissues and predicts a poor prognosis in HCC patients. **a** The mRNA expression level of ACTN1 in 20 paired HCC and NCL tissues was analyzed by real-time qPCR. **b** The protein expression level of ACTN1 in 5 paired HCC and NCL tissues. **c** Immunohistochemical staining of ACTN1 in a tissue microarray containing 180 cases of HCC samples. Scale bar: 50 μm. **d** Representative immunohistochemical images of ACTN1 in HCC, thrombus, NCL and normal liver tissues. Scale bar: 50 μm. **e**, **f** Kaplan-Meier curve analysis of overall survival (OS) and disease-free survival (DFS) in HCC patients based on the expression of ACTN1. **P < 0.01

To investigate the expression pattern of ACTN1 in HCC tissues, we first analyzed ACTN1 mRNA expression in paired HCC and non-cancerous liver (NCL) tissues (*n* = 20) by real-time qPCR. As shown in Fig. 1a, ACTN1 mRNA level had an approximately three-fold increase in HCC tissues compared to NCL tissues. To further determine ACTN1 expression in HCC tissues, western blotting and immunohistochemical staining were performed. Western blotting analysis of 5 independent cases revealed that ACTN1 was found to be markedly increased in HCC tissues compared with NCL tissues (Fig. 1b). Immunohistochemical result confirmed the overexpression pattern of ACTN1 in HCC and thrombus tissues and its cytoplasmic distribution (Fig. 1c).

Next, a tissue microarray containing 157 cases of matched HCC samples and NCL tissues was used to analyze the correlation between ACTN1 expression and corresponding clinicopathological parameters. Immunohistochemical analysis showed that ACTN1 was highly expressed in 69.4% (132/157) of HCC patients (Fig. 1d and e). To evaluate the clinical significance of ACTN1 in HCC, the Chi-square test was used to determine the correlation between ACTN1 expression and corresponding clinicopathologic parameters including age, gender, hepatitis history, gamma-glutamytransferase, alpha-fetoprotein, tumor thrombus, tumor differentiation, vascular invasion, tumor size, and TNM stage. Interestingly, we found that the expression of ACTN1 was closely associated with alpha-fetoprotein level, tumor thrombus, tumor size and TNM stage (Table 1). Moreover, the correlation between ACTN1 expression and clinical follow-up was analyzed by Kaplan-Meier analysis and log-rank test. As displayed in Fig. 1f, high expression of ACTN1 was positively correlated with poor overall survival (OS, *P* = 0.027) in HCC. However, ACTN1 did not predict a poor disease-free survival (DFS; Fig. 1g). By data mining TCGA cohort, we noticed that HCC samples with high ACTN1 expression were prone to have a poor clinical outcome (Fig. 1h).

**Table 1:** Correlations between ACTN1 expression and clinicopathologic parameters in HCC patients

Variable	ACTN1 (n)		
	Low (*n* = 41)	High (*n* = 116)	*P*-value
Age			
≤ 50 years	20	53	0.733
> 50 years	21	63	
Gender			
Female	7	21	0.882
Male	34	95	
Hepatitis history			
Yes	32	91	0.957
No	9	25	
Gamma-glutamytransferase			
≤ 50(U/L)	13	40	0.747
> 50(U/L)	28	76	
*Alpha-fetoprotein			
≤ 20 ng/mL	19	27	0.005
> 20 ng/mL	22	89	
*Tumor thrombus			
Yes	7	41	0.029
No	34	75	
Tumor differentiation			
I-II	15	44	0.878
III	26	72	
Vascular invasion			
Yes	18	36	0.136
No	23	80	
*Tumor size			
≤ 5 cm	35	79	0.033
> 5 cm	6	37	
*TNM stage			
I-II	33	73	0.039
III	8	43	

Pearson’s χ^2^ test was used. The asterisk indicates *P*-values with significant differences

### Genetic silencing of ACTN1 suppresses in vitro cell proliferation of HCC cells

**Fig. 2:**
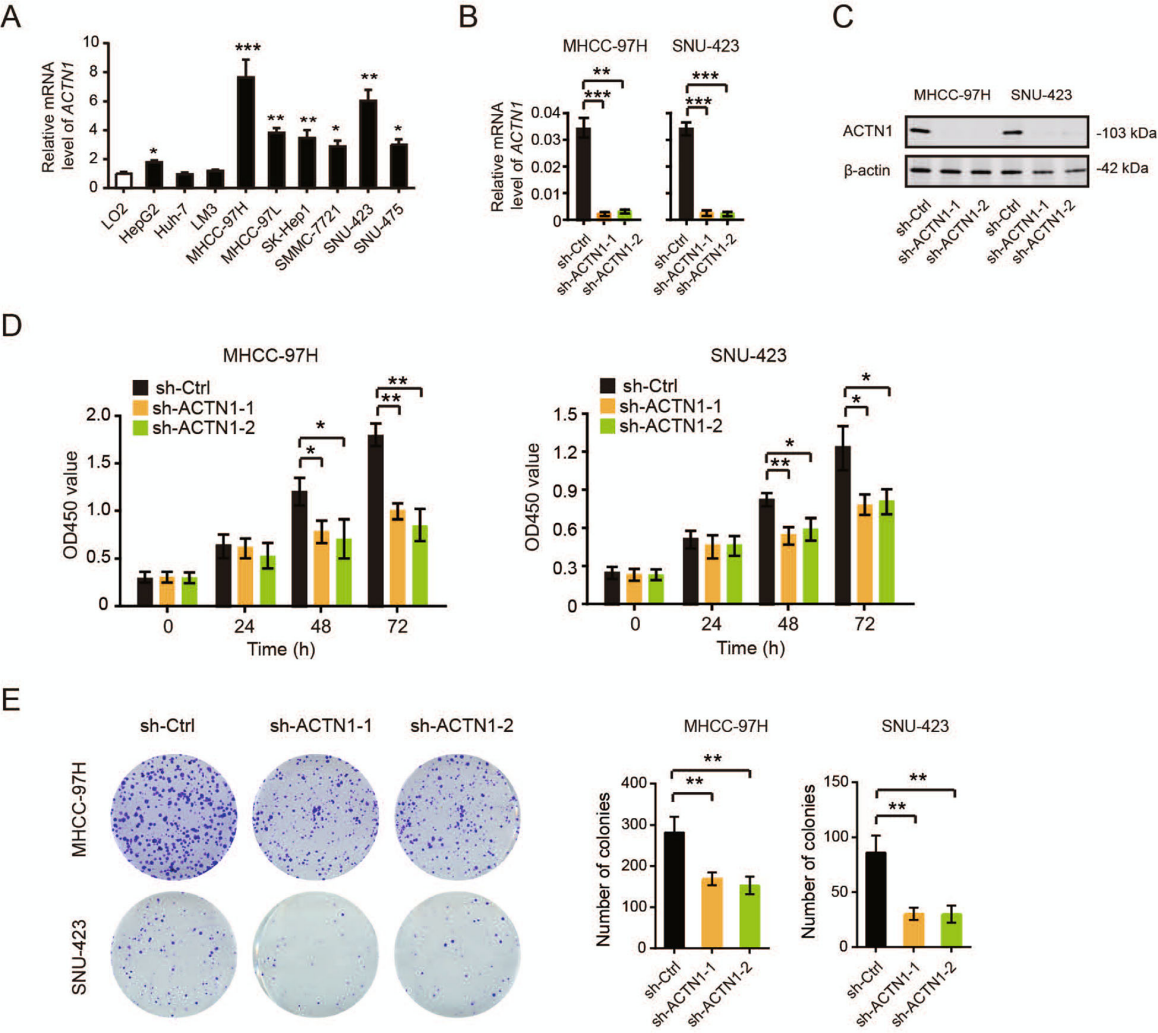
Genetic silencing of ACTN1 suppresses in vitro cell proliferation of HCC cells. **a** The mRNA expression level of ACTN1 in liver cancer cell lines, including HepG2, Huh-7, LM3, MHCC-97H, MHCC-97 L, SK-Hep1, SMMC-7721, SNU-423 and SNU-475 cells. **b** Real-time qPCR analysis of the knockdown efficiency of ACTN1 in MHCC-97H and SNU-423 cells. The experiments were repeated three times. **c** Western blotting analysis of the knockdown efficiency of ACTN1 in MHCC-97H and SNU-423 cells. **d** CCK8 assay of sh-ACTN1 and sh-Ctrl MHCC-97H and SNU-423 cell viability at 0, 24, 48 and 72 h time points. The experiments were repeated three times. **e** Colony formation assay of sh-ACTN1 and sh-Ctrl MHCC-97H and SNU-423 cell viability at 48 h time point. The experiments were repeated three times. *P < 0.05; **P < 0.01; ***P < 0.001

The close relationship between ACTN1 expression and poor clinical outcome indicative of a potential oncogenic role of ACTN1 in HCC, we therefore determined the cellular functions of ACTN1 in HCC cells. Firstly, we detected the mRNA expression of ACTN1 in 9 liver cancer cell lines and the non-malignant LO2 cells. The result showed that ACTN1 was commonly up-regulated in liver cancer cells compared to LO2 cells (Fig. 2a). Notably, ACTN1 had higher expression levels in MHCC-97H and SNU-423 cells, which were further selected for shRNA-mediated loss-of-function study. Through real-time qPCR analysis, we found that two shRNA against ACTN1 successfully silenced ACTN1 expression in MHCC-97H and SNU-423 cells (Fig. 2b). This result was further confirmed by western blotting (Fig. 2c).

Then we investigated the biological function of ACTN1 in the cell proliferation of HCC cells. By CCK8 assay, we found that the cell viability of MHCC-97H or SNU-423 cells was significantly suppressed at 48-h and 72-h time points respectively after ACTN1 was silenced (Fig. 2d). To observe the long-term effect of ACTN1 knockdown on HCC cell proliferation, we carried out plate colony formation assay. Expectedly, the number of colonies was significantly reduced by ACTN1 knockdown in both MHCC-97H and SNU-423 cells (Fig. 2e). Collectively, these data above suggest that ACTN1 is critically involved in the cell proliferation of HCC. Meanwhile, we also detected the effects of ACTN1 knockdown on the migration and F-actin organization of MHCC-97H or SNU-423 cells. It was found that knockdown of ACTN1 could suppress the migration of MHCC-97H or SNU-423 cells (Supplementary Fig. 1). Given that Factin cytoskeleton organization plays crucial roles in cell invasion, we next investigated the effect of ACTN1 knockdown on the cytoskeletal phenotype. As shown in Supplementary 2, disruption of actin fibres was observed in sh-ACTN1 MHCC-97H or SNU-423 cells as revealed by less F-actin cytoskeleton organization at the leading edges of the cells, i.e., in lamellipodia and filopodia.

### Knockdown of ACTN1 suppresses in vivo tumor growth of HCC

**Fig. 3:**
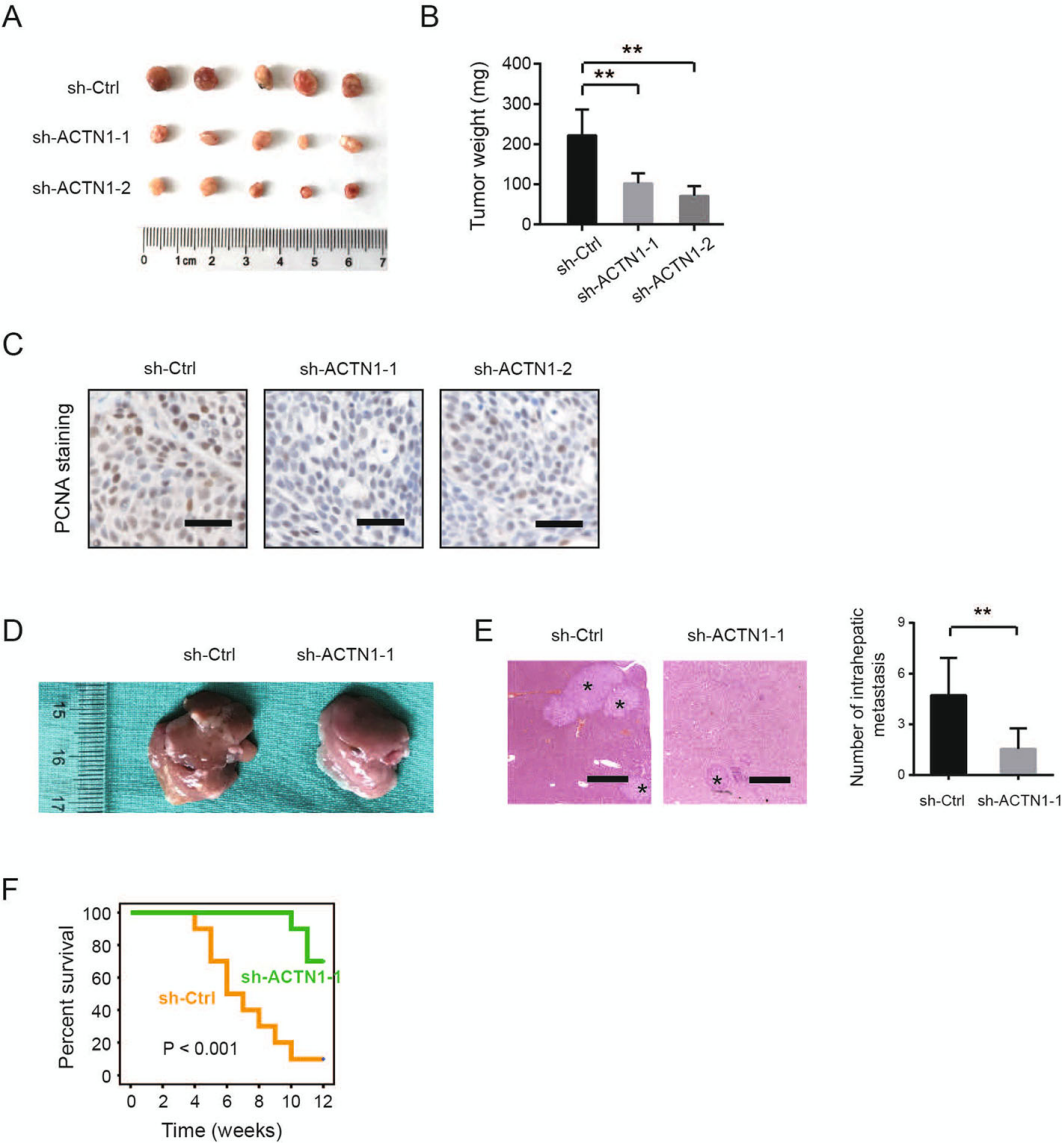
Knockdown of ACTN1 suppresses in vivo tumor growth of HCC. **a** Images of tumors from mice subcutaneously inoculated with sh-ACTN1–1, sh-ACTN1–2 or sh-Ctrl MHCC-97H cells (*n* = 5). **b** Statistical analysis of tumor weight from sh-ACTN1–1, sh-ACTN1–2 and sh-Ctrl groups. **c** IHC analysis of PCNA staining in the tumors of sh-ACTN1–1, sh-ACTN1–2 and sh-Ctrl groups. Scale bar: 50 μm. **d** Representative images of liver tissues from mice orthotopically inoculated with sh-ACTN1–1 or sh-Ctrl MHCC-97H cells. **e** H&E staining in liver tissues from sh-ACTN1–1 or sh-Ctrl group. Statistical analysis of intrahepatic metastatic foci is shown at right. Scale bar: 500 μm. **f** Kaplan-Meier survival curve analysis of the mice survival in the sh-ACTN1–1 and sh-Ctrl group. **P < 0.01

To investigate the growth-promoting effect of ACTN1 in vivo, we generated a subcutaneous xenograft model by injection of sh-ACTN1–1, sh-ACTN1–2 and sh-Ctrl MHCC-97H cells into nude mice. After 4 weeks, mice were sacrificed and tumor tissues were dissected (Fig. 3a). As a result, we found that ACTN1 knockdown significantly retarded tumor growth as revealed by reduced tumor weight (Fig. 3b). By IHC staining, we also found that the proliferation index PCNA expression in the sh-ACTN1 group was markedly lower than that in the vehicle group (Fig. 3c).

To assess intrahepatic HCC tumor growth, we orthotopically injected sh-ACTN1 and control MHCC-97H cells into nude mice. Four weeks later, mice were sacrificed and liver tissues were dissected (Fig. 3d). Histological H&E staining of the liver tissues showed that mice transplanted with sh-ACTN1–1 cells had lesser intrahepatic tumor nodules than those transplanted with sh-Ctrl cells (Fig. 3e). In another cohort, mice survival time in the sh-ACTN1–1 group was significantly increased compared with the sh-Ctrl group (Fig. 3f). Collectively, these findings above suggest that ACTN1 is profoundly implicated in the tumor growth of HCC cells.

### ACTN1 regulates Hippo signaling activity and Rho GTPases activities

**Fig. 4:**
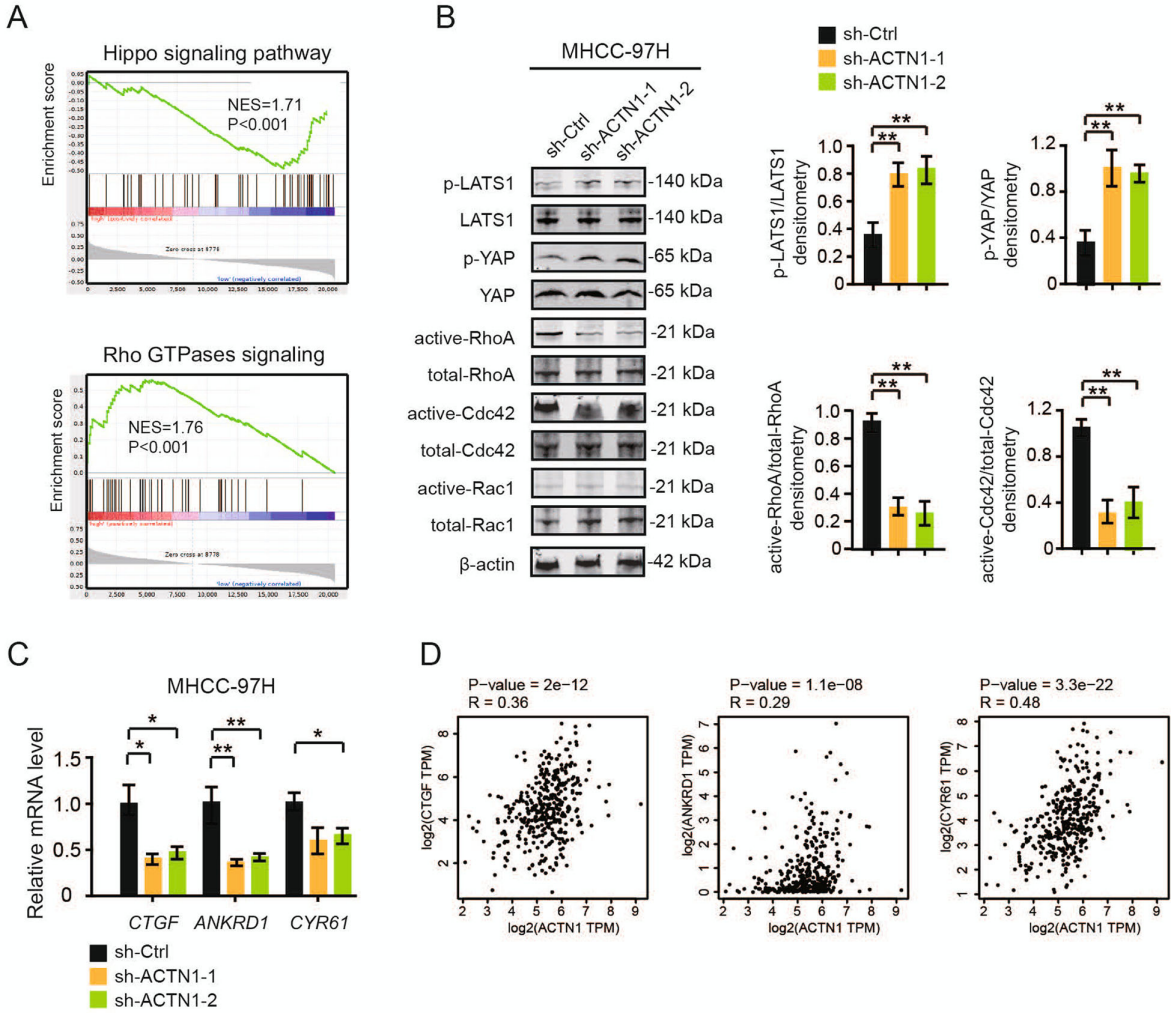
ACTN1 regulates Hippo signaling activity and Rho GTPases activities. **a** The Cancer Genome Atlas (TCGA) RNA sequencing data of HCC cohort were downloaded and divided into two ACTN1-high and ACTN1-low groups. Gene set enrichment analysis (GSEA) was then preformed. False discovery rate (FDR) was set at 0.25. **b** Western blotting analysis of phospho-LATS1, LATS1, phospho-YAP and YAP in sh-ACTN1–1 and sh-Ctrl MHCC-97H cells. The activities of RhoA, Cdc42 and Rac1 were measured by pull-down assays in sh-ACTN1–1 and sh-Ctrl MHCC-97H cells. Statistical analyses of densitometry were shown at right. The experiments were repeated three times. **c** Real-time qPCR analysis of the mRNA levels of CTGF, ANKRD1 and CYR61 in sh-ACTN1–1 and sh-Ctrl MHCC-97H cells. The experiments were repeated three times. **d** Correlation analysis of ACTN1 expression with YAP target genes in the TCGA cohort. *P < 0.05; **P < 0.01

To uncover the underlying molecular mechanism of the association of ACTN1 with HCC, we analyzed ACTN1-associated gene expression profiles by using the RNA sequencing data of HCC patients in the TCGA cohort. Based on the median value of ACTN1, HCC patients were divided into two groups: ACTN1-high and ACTN1-low. Gene set enrichment analysis revealed that ACTN1 was closely related with Hippo signaling and Rho GTPases signaling (Fig. 4a). To check whether ACTN1 affects Hippo signaling, we performed western blotting analyses and found that knockdown of ACTN1 in MHCC-97H cells significantly increased the phosphorylated level of LATS1 and YAP (Fig. 4b), suggesting a inhibitory role of ACTN1 on Hippo signaling. Additionally, ACTN1-silenced and control MHCC-97H cells were allowed to serum starvation for 24 h and subjected for analysis of activities of Rho GTPases by pull-down assays. Intriguingly, RhoA and Cdc42 activities were significantly downregulated by ACTN1 knockdown, while Rac1 activity remained unchanged (Fig. 4b).

Moreover, we also analyzed the mRNA levels of three canonical YAP target genes, including connective tissue growth factor (CTGF), ankyrin repeat domain 1 (ANKRD1), and cysteine rich angiogenic inducer 61 (CYR61). Real-time qPCR result showed that the mRNA level of CTGF, ANKRD1 and CYR61 were significantly reduced in ACTN1-silenced MHCC-97H cells (Fig. 4c). By data mining the HCC samples in TCGA cohort, we also noticed a close correlation between ACTN1 expression and these YAP target genes (Fig. 4d).

### ACTN1 competitively interacts with MOB1 to regulate Hippo signaling activity

**Fig. 5:**
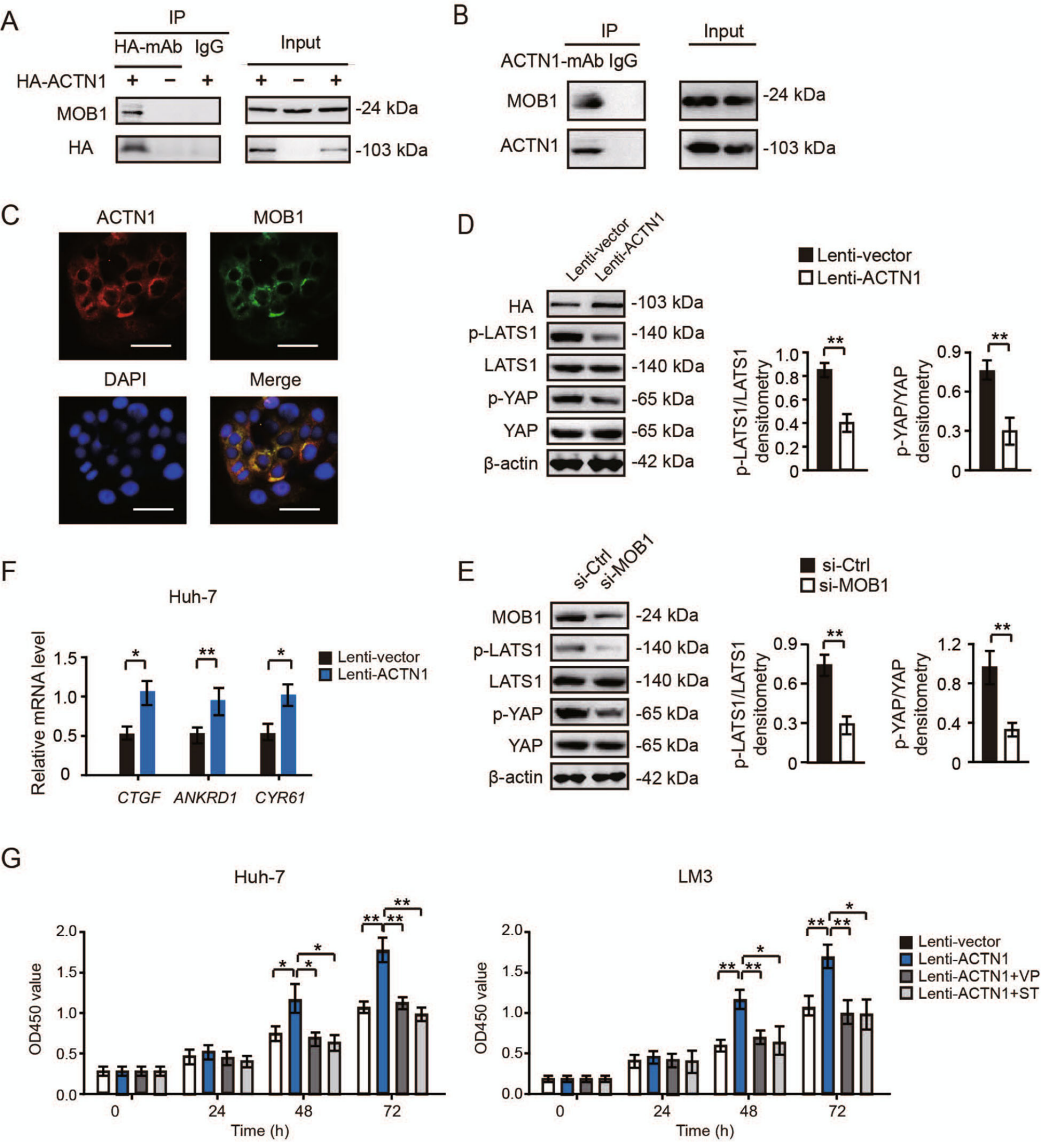
ACTN1 competitively interacts with MOB1 to regulate Hippo signaling activity. **a** Co-immunoprecipitation analysis of the physical interaction between ACTN1 and MOB1 in the Huh-7 cells. **b** Co-immunoprecipitation analysis of the interaction between ACTN1 and MOB1 in the MHCC-97H cells. **c** Co-immunofluorescence analysis of ACTN1 and MOB1 expression in the Huh-7 cells. Scale bar: 10 μm. **d** Western blotting analysis of phospho-LATS1, LATS1, phospho-YAP and YAP in lenti-ACTN1 and lenti-vector Huh-7 cells. Statistical analyses of densitometry were shown at right. The experiments were repeated three times. **e** Western blotting analysis of effect of MOB1 knockdown on the activity of Hippo signaling pathway. Statistical analyses of densitometry were shown at right. The experiments were repeated three times. **f** Real-time qPCR analysis of the mRNA levels of CTGF, ANKRD1 and CYR61 in lenti-ACTN1 and lenti-vector Huh-7 cells. The experiments were repeated three times. **g** CCK8 experiment of the cell viability of lenti-ACTN1 and lenti-vector Huh-7 or LM3 cells upon MOB1 knockdown in the presence or absence of 50 nM Verteporfin or 50 nM Super-TDU (1–31) (inhibitors of YAP-TEAD complex). The cell viability of Huh-7 and LM3 cells was analyzed after 24, 48 and 72 h. The experiments were repeated three times. *P < 0.05; **P <  0.01

Next, we investigated whether ACTN1 affects Hippo signaling via directly association with the components of the Hippo signaling pathway by co-immunoprecipitation experiments. To achieve this, we overexpressed ACTN1 in Huh-7 cells, in which ACTN1 had a lower endogenous expression level. Huh-7 cells were transfected with HA-tagged ACTN1 or vector control. Interestingly, we found that immunoprecipitates of ACTN1 from Huh-7 cells contained MOB1, an accessory protein of LATS1 (Fig. 5a). We further confirmed the interaction between ACTN1 and MOB1 by performing co-immunoprecipitation analysis in MHCC-97H cells which has a high level of endogenous ACTN1 (Fig. 5b). By immunofluorescence staining, the co-localization of ACTN1 and MOB1 was observed in MHCC-97H cells (Fig. 5c). Moreover, we found that depletion of MOB1 could rescue the inhibitory effects of ACTN1 knockdown on the proliferation of MHCC-97H and SNU-423 cells, and increase the mRNA levels of CTGF, ANKRD1 and CYR61 which were suppressed by ACTN1 knockdown (Supplementary Fig. 3).

Moreover, overexpression of ACTN1 significantly suppressed the phosphorylation of LATS1 or YAP in Huh-7 cells (Fig. 5d). Meanwhile, we also knocked down MOB1 in Huh-7 cells, and found that the phosphorylation of LATS1 or YAP was inhibited in MOB1-silenced Huh-7 cells (Fig. 5e). These findings suggest that ACTN1 may affect Hippo signaling via physical interaction with MOB1. Additionally, we found that the mRNA level of CTGF, ANKRD1 and CYR61 were significantly enhanced in ACTN1-overexpressed Huh-7 cells by real-time qPCR (Fig. 5f). To determine whether ACTN1-mediated growth-promoting effect is dependent on Hippo signaling, we detected Huh7 and LM3 cell viability upon ACTN1 overexpression in the presence or absence of Verteporfin (VP) or Super-TDU (1–31) (ST), which are inhibitors of the YAP-TEAD complex. As was expected, VP and ST could largely compromise the increased cell viability induced by ACTN1 in Huh-7 or LM3 cells proliferation (Fig. 5g).

**Fig. 6:**
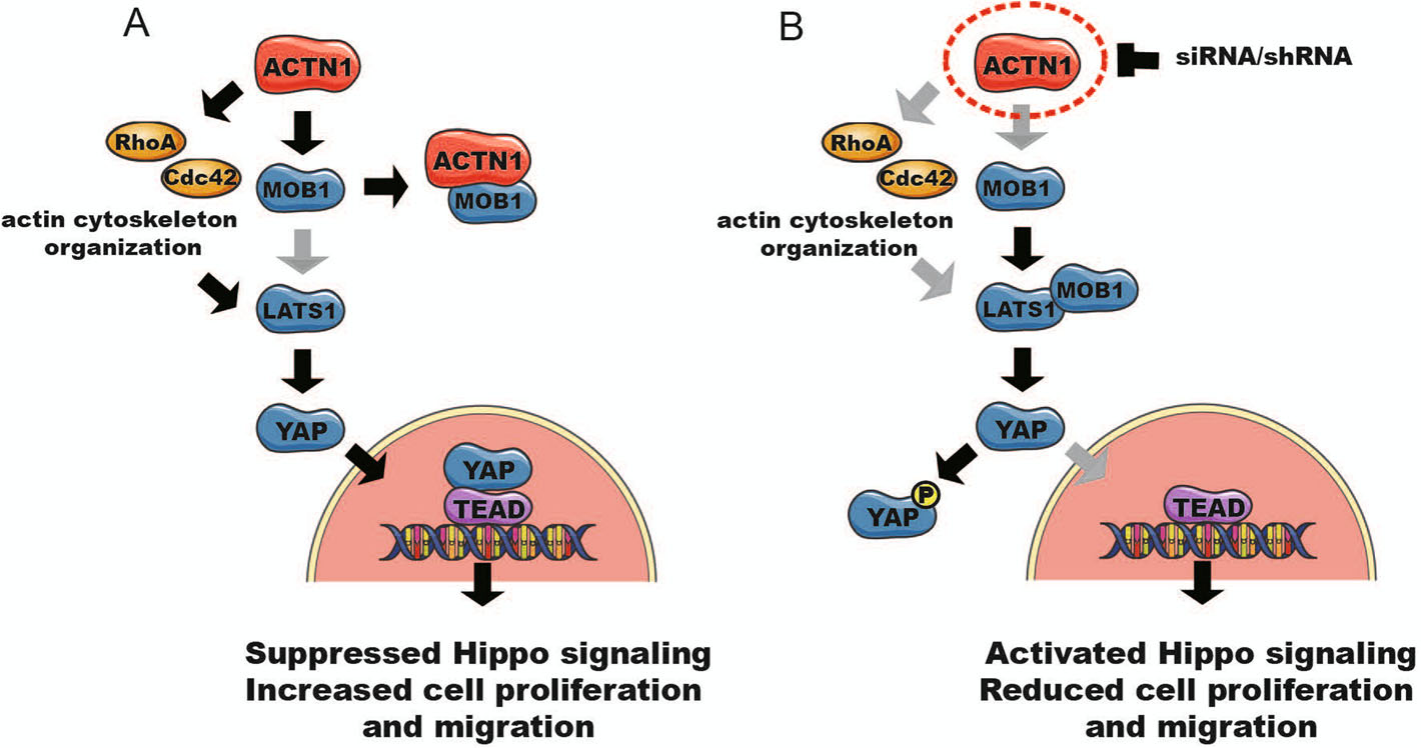
The molecular mechanism of ACTN1 in regulating Hippo signaling and cell proliferation of HCC. **a** ACTN1 competitively interacts with MOB1 and decreases the phosphorylation of LATS1/YAP, and ultimately suppresses Hippo signaling and promotes the proliferation/migration of HCC. **b** If ACTN1 is knocked down, MOB1 can interact with LATS1, then increase the phosphorylation of LATS1/YAP and activate Hippo signaling, which lead to the inhibitory effects on the tumor growth of HCC

Altogether, these results suggested that ACTN1 could competitively interact with MOB1 and decrease the phosphorylation of LATS1/YAP, thus suppress Hippo signaling and promote the cell proliferation of HCC (Fig. 6a). When ACTN1 was knocked down, MOB1 could interact with LATS1, then increase the phosphorylation of LATS1/YAP and activate Hippo signaling, which led to the inhibitory effects on the tumor growth of HCC (Fig. 6b).

## Discussion

ACTN1 has been proved to be associated with the poor prognosis in breast cancer, oral squamous cell carcinoma, and acute lymphoblastic leukemia [26–28]. Targeting ACTN1 by Oroxylin A could remodel stromal microenvironment and restrain breast cancer metastasis [29]. In this study, we for the first time investigated the detailed biological functions and related mechanism of ACTN1 in HCC. It was found that the expression of ACTN1 is closely related with tumor size, TNM stage, and patient prognoses. The in vitro and in vivo experiments revealed that ACTN1 is crucial for the cell proliferation and tumor growth of HCC. All of these data suggested that ACTN1 plays an important role in the development of HCC.

During the progression of cancer, Hippo signaling pathway plays diverse important roles [10, 30]. It was reported that the Hippo pathway is profoundly implicated in modulating various cancer-related malignant phenotypes, including cell proliferation, invasion and metastasis of tumor cells [30, 31]. In HCC, Hippo signaling is recognized as a major tumor suppressive pathway by inhibiting hepatocyte proliferation, survival and HCC formation [32, 33]. Activation of YAP/TAZ transcription activity is essential for HCC proliferation [34, 35]. Hippo signaling inhibits YAP/TAZ transcription activity via activating LATS1/2 kinases that directly phosphorylates YAP/TAZ, resulting in YAP/TAZ cytoplasmic retention and subsequent degradation [11, 13]. In the current study, our findings revealed that knockdown of ACTN1 increases the phosphorylation level of YAP and LATS1, and thus restrained them in the cytoplasm where they lost their transcriptional activation. Furthermore, these results were confirmed by detecting the alteration of canonical YAP target genes. YAP/TAZ is emerged as novel therapeutic targets in cancers [36, 37]. Therefore, targeting ACTN1-mediated YAP activation might provide a new avenue for HCC treatment. Indeed, blocking the YAP-TEAD complex is sufficient to inhibit ACTN1-mediated growth-promoting effect. Alpha-actinin is responsible for the organization of microfilaments in contractile filaments or stress fibers. Indeed, depletion of ACTN1 leads to inhibition of the actin regulators RhoA and CDC42. Moreover, depletion of ACTN1 affects the organization of F-actin of HCC cells. Thus, a Hippo-independent molecular mechanism by which ACTN1 promotes HCC cell migration might exist.

Moreover, in this research we further found that ACTN1 could directly interact with MOB1, which may lead to the loss of combination of MOB1 with LATS1, thus decreased the phosphorylation of LATS1/YAP and suppressed Hippo signaling. Identifying the interaction between ACTN1 and MOB1 provides a direct evidence for the important role of ACTN1 in the regulation of Hippo signaling. Also, we found that ACTN1 overexpression-induced HCC cell proliferation was largely abrogated by the inhibitors of the YAP-TEAD complex, indicating that ACTN1-induced HCC cell proliferation are dependent on the context of inhibition of Hippo signaling. Notably, ACTN1 is also involved in the focal adhesion kinase-Src complex formation [38], destabilization of E-cadherin-based adhesions [28], and luminogenesis [39]. Therefore, more works are needed to uncover whether these ACTN1-related activities are implicated in HCC development and progression. Previously, ACTN1 has been reported to play contrasting roles in RhoA signaling of astrocytoma cells [40]. Here, we identified a positive regulatory role of ACTN1 in Rho GTPases in HCC cells. However, the detailed molecular mechanism underlying ACTN1-mediated Rho GTPases activities is warranted for further investigations.

## Conclusions

In conclusion, in this study we found that ACTN1 is crucial for the cell proliferation and tumor growth of HCC. ACTN1 suppresses the Hippo signaling pathway in HCC by interacting with MOB1, contributing to the phosphorylation of LATS1/YAP and increased HCC cell proliferation. ACTN1 may be acted as a novel Hippo signaling modulator in HCC and is a potential therapeutic target for HCC.

## Supplementary Information

**Additional file 1: Supplementary Figure 1.** Silencing of ACTN1 suppresses in vitro cell migration of HCC cells. (A) The images of migrated MHCC-97H cells in sh-ACTN1–1, sh-ACTN1–2 and sh-Ctrl groups. (B) The images of migrated SNU-423 cells in sh-ACTN1–1, sh-ACTN1–2 and sh-Ctrl groups. Scale bar: 100 μm. **P < 0.01.

**Additional file 2: Supplementary Figure 2.** Silencing of ACTN1 suppresses F-actin organization of HCC cells. (A) The images of phalloidin stained MHCC-97H cells in sh-ACTN1–1, sh-ACTN1–2 and sh-Ctrl groups. (B) The images of phalloidin stained SNU-423 cells in sh-ACTN1–1, sh-ACTN1–2 and sh-Ctrl groups. Scale bar: 50 μm.

**Additional file 3: Supplementary Figure 3.** Depletion of MOB1 rescues the inhibitory effects of ACTN1 knockdown on the proliferation of MHCC-97H or SNU-423 cells and mRNA levels of canonical YAP target genes. (A) CCK8 assay of sh-Ctrl, sh-ACTN1 and sh-ACTN1 MHCC-97H cell viability in the presence or absence of si-MOB1at 0, 24, 48 and 72 h time points. (B) CCK8 assay of sh-Ctrl, sh-ACTN1 and sh-ACTN1 SNU-423 cell viability in the presence or absence of si-MOB1 at 0, 24, 48 and 72 h time points. (C) Real-time qPCR analysis of the mRNA levels of CTGF, ANKRD1 and CYR61 in sh-Ctrl, sh-ACTN1 and sh-ACTN1 MHCC-97H cells in the presence or absence of si-MOB1. The experiments were repeated three times. *P < 0.05; **P < 0.01.

## Data Availability

All data generated or analyzed during this research are included in this manuscript.

## Abbreviations

ACTN1: α-actinin 1
HCC: Hepatocellular carcinoma
NCL: Non-cancerous liver
OS: Overall survival
DFS: Disease-free survival
YAP: Yes Associated Protein
LATS1: Large Tumor Suppressor Kinase 1
CTGF: Connective Tissue Growth Factor
ANKRD1: Ankyrin Repeat Domain 1
CYR61: Cysteine Rich Angiogenic Inducer 61
