# Correction to: ACTN1 supports tumor growth by inhibiting Hippo signaling in hepatocellular carcinoma

**DOI:** 10.1186/s13046-021-01924-8

**Published:** 2021-04-12

**Authors:** Qian Chen, Xiao-Wei Zhou, Ai-Jun Zhang, Kang He

**Affiliations:** 1 Reproductive Medical Center, Department of Obstetrics and Gynecology of Ruijin Hospital, School of Medicine, Shanghai Jiaotong University, 197 Ruijin 2nd Road, Shanghai, 200025 China; 2 Department of Liver Surgery, Renji Hospital, School of Medicine, Shanghai Jiaotong University, 160 Pujian Road, Shanghai, 200127 China

**Correction to: J Exp Clin Cancer Res 40, 23 (2021)**

**https://doi.org/10.1186/s13046-020-01821-6**

Following publication of the original article [1], the authors identified minor errors in image-typesetting in Fig. 1; specifically in panels presented in Fig 1c, d, f and g. The corrected figure and caption are given below.

**Fig. 1:**
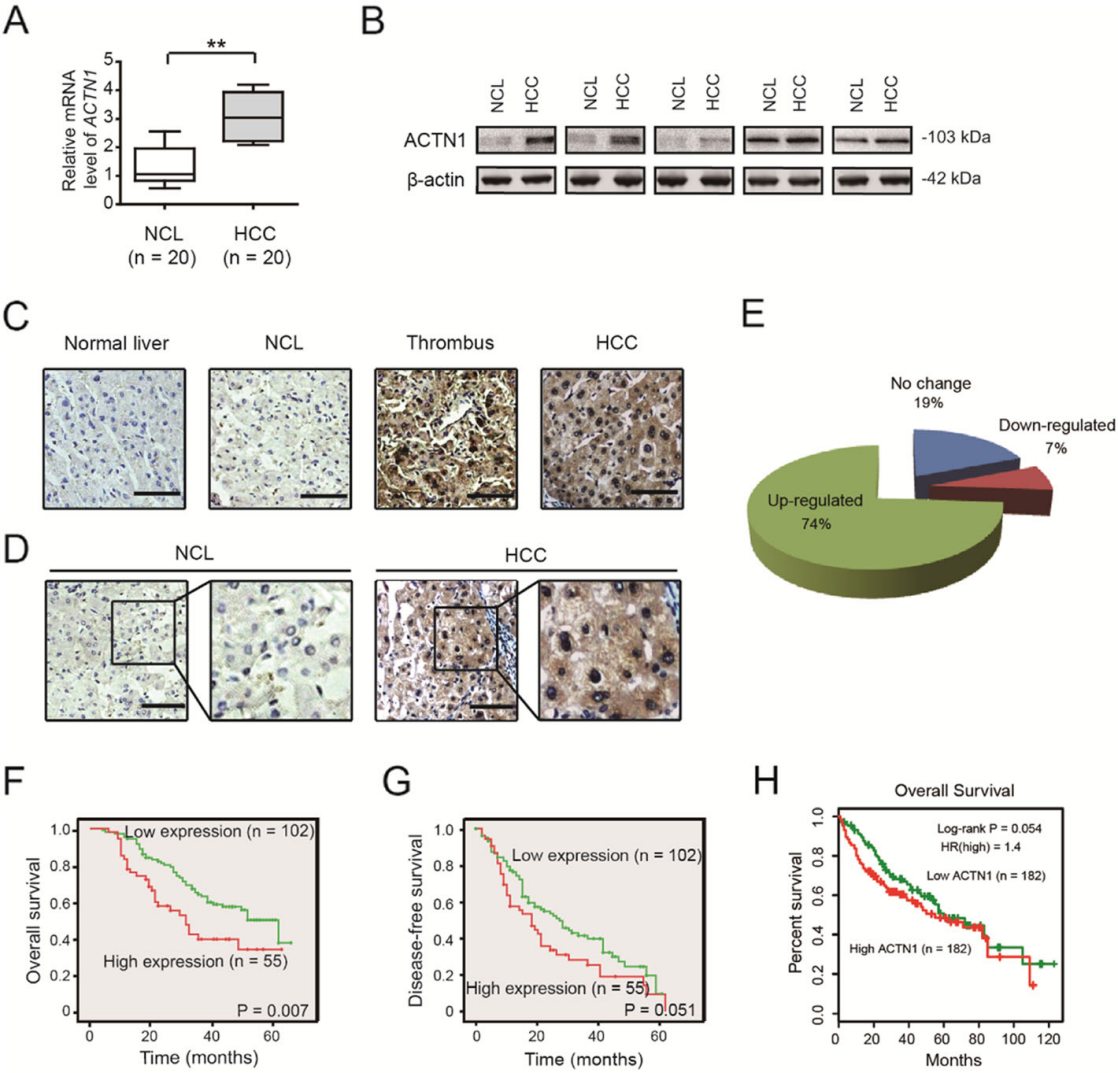
ACTN1 is highly expressed in HCC tissues and predicts a poor prognosis in HCC patients. **a** The mRNA expression level of ACTN1 in 20 paired HCC and NCL tissues was analyzed by real-time qPCR. **b** The protein expression level of ACTN1 in 5 paired HCC and NCL tissues. **c** Representative immunohistochemical images of ACTN1 in HCC, thrombus, NCL and normal liver tissues. Scale bar: 50 μm. **d** Immunohistochemical staining of ACTN1 in a tissue microarray containing 157 cases of HCC samples. Scale bar: 50 μm. **e** The expression of ACTN1 was up-regulated in 74% of HCC patients. **f-g** Kaplan-Meier curve analysis of overall survival (OS) and disease-free survival (DFS) in HCC patients based on the expression of ACTN1. **h** Kaplan-Meier curve analysis of OS in TCGA cohort based on the expression of ACTN1. **P < 0.01

In addition, sections of the mainbody text have been corrected in light of the above. In the ‘Results’ section, under the heading ‘Highly expressed ACTN1 predicts a poor clinical outcome in HCC patients’, the following sentences have been corrected (emphasis given to affected areas):

“Immunohistochemical analysis showed that ACTN1 was highly expressed in **69.4% (132/157)** of HCC patients (Fig. 1d and e).” has been corrected to: “Immunohistochemical analysis showed that ACTN1 was highly expressed in **74% (116/157)** of HCC patients (Fig. 1d and e).

“As displayed in Fig. 1f, high expression of ACTN1 was positively correlated with poor overall survival (OS, ***P*** **= 0.027**) in HCC.” Has been corrected to: “As displayed in Fig. 1f, high expression of ACTN1 was positively correlated with poor overall survival (OS, ***P*** **= 0.007**) in HCC.”

The correction does not have any effect on the results or conclusions of the paper. The original article has been corrected.
