# Supplementary figures and images for: ACTN1 supports tumor growth by inhibiting Hippo signaling in hepatocellular carcinoma
Source: J Exp Clin Cancer Res. 2021 Jan 7;40:23. doi: 10.1186/s13046-020-01821-6 (PMC7791991; doi:10.1186/s13046-020-01821-6)

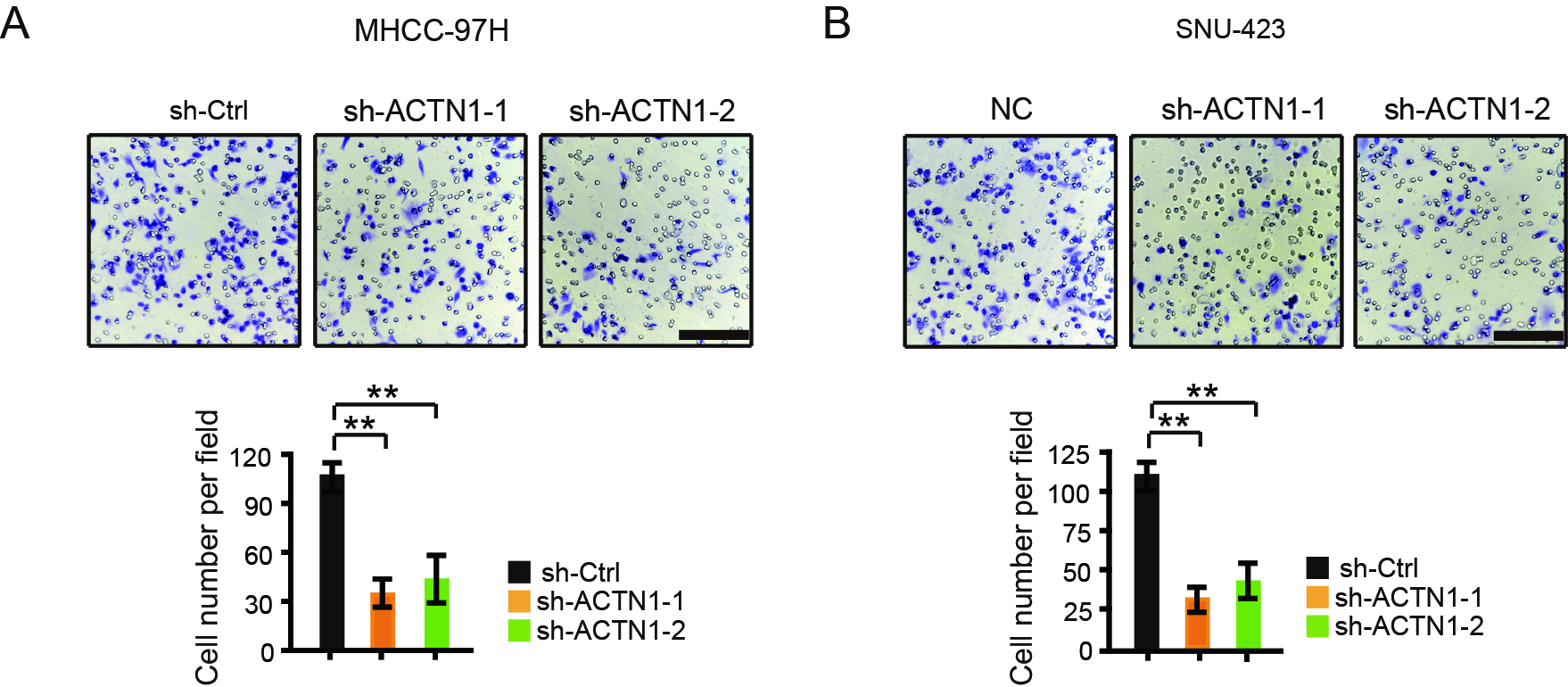

Supplement: Supplementary file 1 — Additional file 1: Supplementary Figure 1. Silencing of ACTN1 suppresses in vitro cell migration of HCC cells. (A) The images of migrated MHCC-97H cells in sh-ACTN1–1, sh-ACTN1–2 and sh-Ctrl groups. (B) The images of migrated SNU-423 cells in sh-ACTN1–1, sh-ACTN1–2 and sh-Ctrl groups. Scale bar: 100 μm. **P < 0.01. [file 13046_2020_1821_MOESM1_ESM.tif]

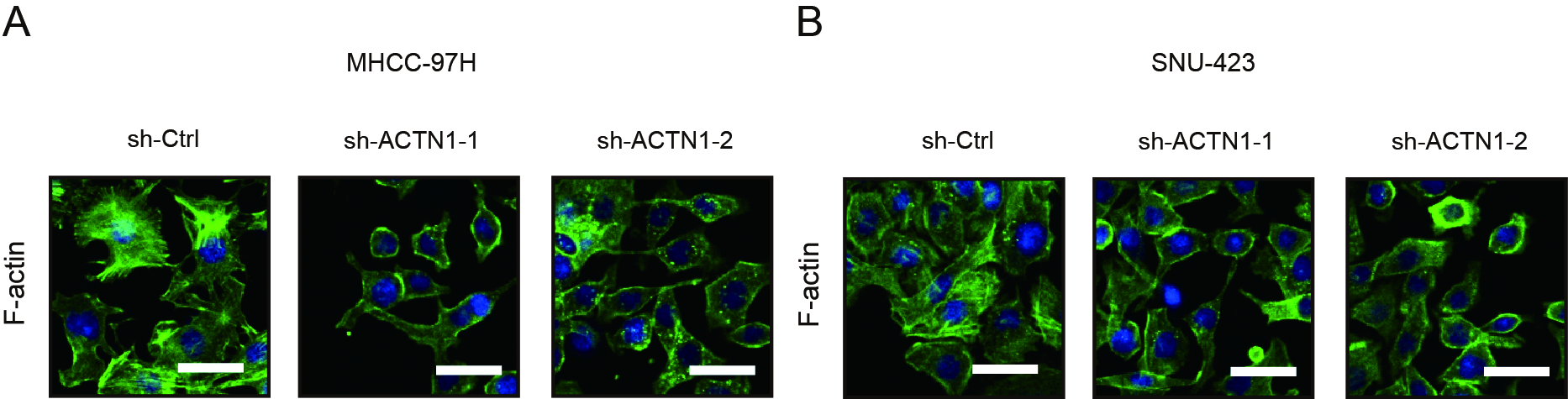

Supplement: Supplementary file 2 — Additional file 2: Supplementary Figure 2. Silencing of ACTN1 suppresses F-actin organization of HCC cells. (A) The images of phalloidin stained MHCC-97H cells in sh-ACTN1–1, sh-ACTN1–2 and sh-Ctrl groups. (B) The images of phalloidin stained SNU-423 cells in sh-ACTN1–1, sh-ACTN1–2 and sh-Ctrl groups. Scale bar: 50 μm. [file 13046_2020_1821_MOESM2_ESM.tif]

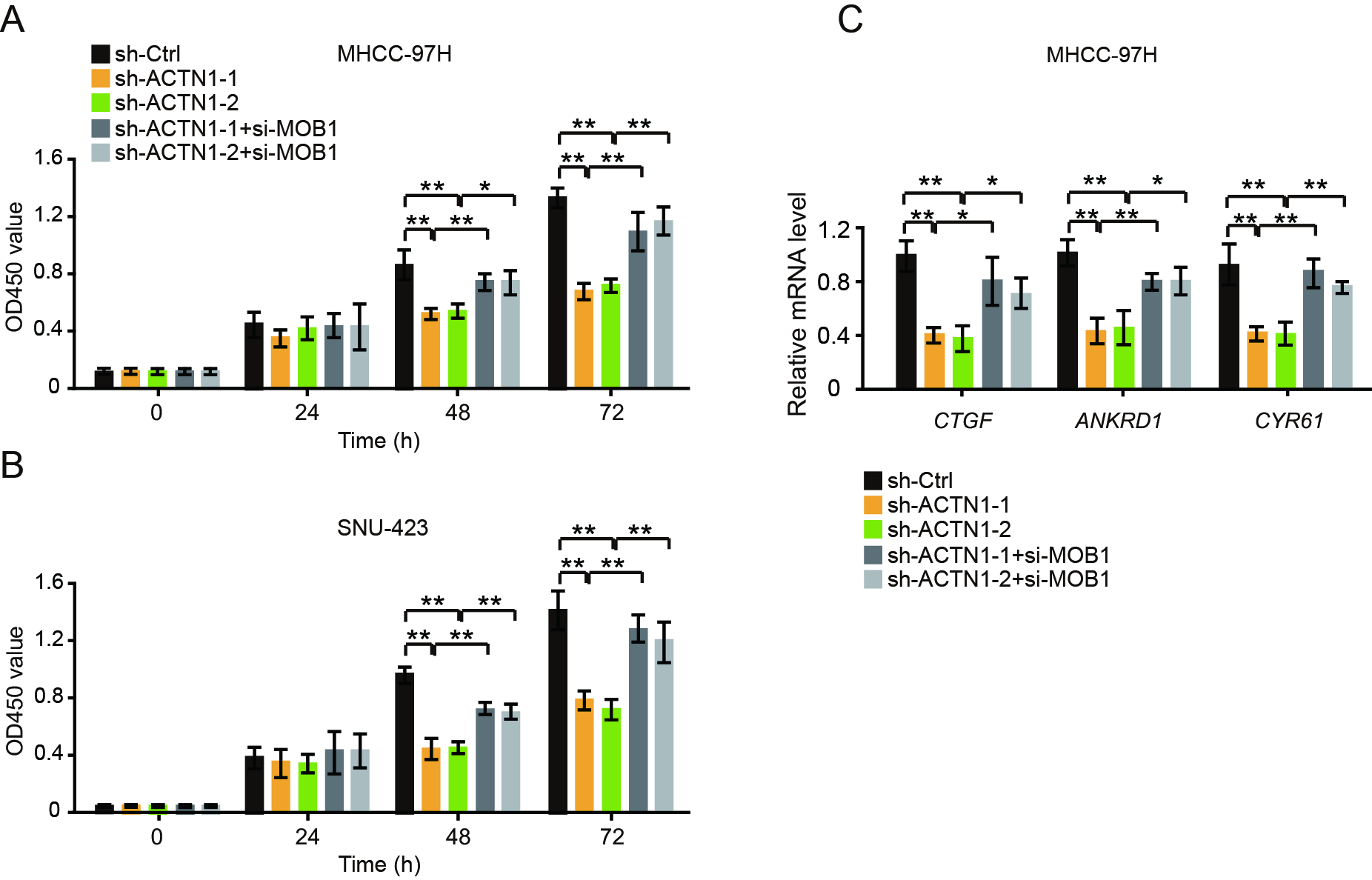

Supplement: Supplementary file 3 — Additional file 3: Supplementary Figure 3. Depletion of MOB1 rescues the inhibitory effects of ACTN1 knockdown on the proliferation of MHCC-97H or SNU-423 cells and mRNA levels of canonical YAP target genes. (A) CCK8 assay of sh-Ctrl, sh-ACTN1 and sh-ACTN1 MHCC-97H cell viability in the presence or absence of si-MOB1at 0, 24, 48 and 72 h time points. (B) CCK8 assay of sh-Ctrl, sh-ACTN1 and sh-ACTN1 SNU-423 cell viability in the presence or absence of si-MOB1 at 0, 24, 48 and 72 h time points. (C) Real-time qPCR analysis of the mRNA levels of CTGF, ANKRD1 and CYR61 in sh-Ctrl, sh-ACTN1 and sh-ACTN1 MHCC-97H cells in the presence or absence of si-MOB1. The experiments were repeated three times. *P < 0.05; **P < 0.01. [file 13046_2020_1821_MOESM3_ESM.tif]
